# Liver lipoma: a case report

**DOI:** 10.1259/bjrcr.20150467

**Published:** 2016-11-03

**Authors:** Guglielmo Manenti, Eliseo Picchi, Antonella Castrignanò, Massimo Muto, Marco Nezzo, Roberto Floris

**Affiliations:** Department of Diagnostic and Molecular Imaging, Interventional Radiology and Radiotherapy, Fondazione PTV Policlinico Tor Vergata, University of Rome “Tor Vergata”, Rome, Italy

## Abstract

Liver lipoma is a rare benign mesenchymal tumour without malignant degeneration. Lesions may be asymptomatic, nevertheless they may sometimes cause abdominal pain depending upon the size. Usually liver lipomas are an incidental finding during radiological examinations performed for other reasons, and there is no evidence of familiar genetic cluster or predisposing factors but it seems to have a strong association with impaired lipidic profile. In this report, we describe the case of a 72 year old female with a giant liver lipoma observed during an ultrasound examination. The clinical examination was completed with CT and MRI scans. Features of the lesion such as negative attenuation values on multiphasic CT examination and MRI signal drop-out on *T*_2_ spectral presaturation with inversion recovery sequences, with no enhancement after administration of contrast medium, are suggestive for this kind of benign neoplasm. The purpose of this report is provide an anthological case of liver lipoma, helping to define the diagnostic features with imaging techniques.

## Case presentation

**Figure 1.**
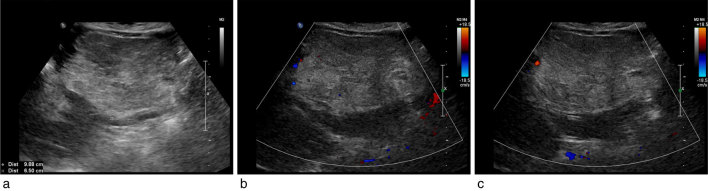
Abdominal ultrasound shows iso-hyperechoic (a) lesion in the left lobe of the liver and its Doppler analysis (b and c) that shows a peripheral pattern of vascularisation.

A 72 year old female, with no significant past medical history, was sent to our hospital by her primary care physician for vague abdominal pain and dyspepsia. The patient had no history of alcohol abuse neither abdominal operation. We performed an abdominal ultrasonography, which revealed a large and well-defined mass of mixed echogenicity (iso-hyperechoic), with posterior attenuation, in the left lobe of the liver (Figure 1a). The maximum axial diameter of the mass was about 10 cm, and a colour Doppler modality has been used to study the lesion showing a perilesional vascularisation (Figure 1b, c). Gallbladder lythiasis was detected, with no dilatation of biliary system; there were no signs of cholecystitis. There were no splenomegaly or ascites. Laboratory studies including the blood cells count and liver function tests were within the limits, only triglycerides and glucose were altered, with a body mass index of 30.8. Hepatitis serology and tumour markers (e.g. alpha-fetoprotein, protein induced by vitamin K absence/antagonist II, carcinoembryonic antigen and carbohydrate antigen 19.9) were negative.

## Differential diagnosis 

Adenoma with fat component, angiomyolipoma, metastases from liposarcoma, fatty changes in hepatocellular carcinoma.

## Investigation and image findings

**Figure 2.**
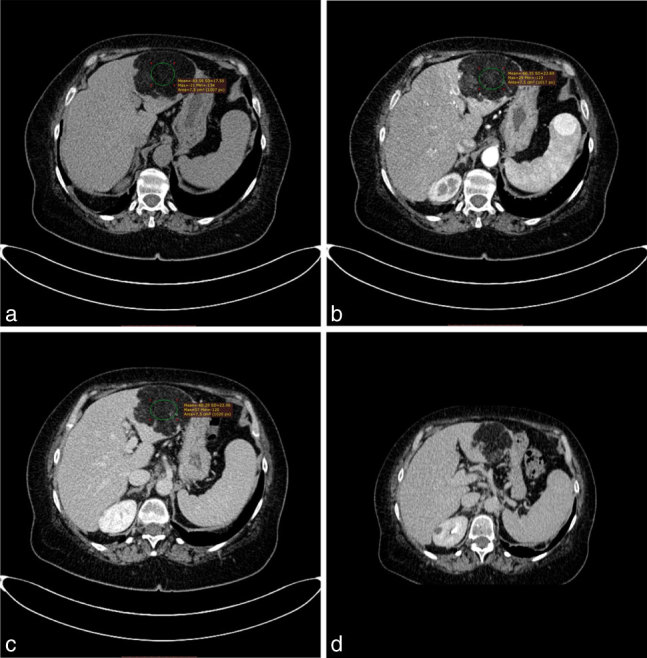
CT examination with contrast medium. (a) TC baseline, (b) arterial phase; (c) portal phase; (d) delayed stage coronal acquisition with axial reconstruction. It could be noted that the lesion has a similar attenuation of fat and does not show significant enhancement in subsequent phases of the study. Please note the presence of a splenic angioma. A region of interest (green ring in the figures), was placed on lesion and it shows the HU attenuation changes in different phases of the CT examination. The HU values do not change significantly in the different phases of acquisition. HU, Hounsfield.

**Figure 3.**
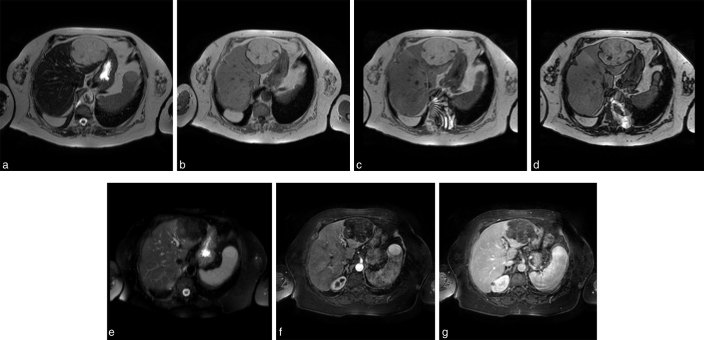
MRI scans in a patient with liver lipoma. (a) axial *T*_2_ weighted image, (b) axial *T*_1_ weighted image; (c) axial in-phase image; (d) axial out-phase image; (e) axial *T*_2_ SPIR image; (f, g) Gadolinium-enhanced fat-suppressed *T*_1_ weighted MRI scan by using THRIVE sequence, early (f at 25^″^ after administration of i.v. contrast medium) and delayed (g at 150^″^ after administration of i.v. contrast medium) phases. Please notice how lesion is hyperintense on *T*_1_ and *T*_2_ weighted images, without signal drop-out in the core on *out-phase sequences* owing to few water protons to cancel out the fat signal on the same voxel. There is only a signal drop-out on *out-of-phase* sequence at the edge of the lesion because in these periferical voxel there is almost the same concentrations of fat and water protons, which cancel out the fat signal. On *T*_2_ SPIR the lesion has signal suppression while in THRIVE sequences the lesion does not show a significant enhancement confirming the benign assumption. The subtle artefact on anteriorly in the left and right sides is caused by an P-reduction = 2 because the patients was uncooperative and claustrophobic. SPIR, spectral presaturation with inversion recovery; THRIVE, *T*_1_ high resolution isotropic volume excitation.

A contrast-enhanced CT scan has been planned and performed within few days. Plain CT scan showed a round, non-infiltrating lesion, with lobulated and defined margins. The lesion was homogenous, with a fat-like tissue and thin septa, with no significant contrast-enhancement both in early and delayed phases (Figure 2a–d). A region of interest, of 7 mm^2^, was placed in the core of the lesion and the attenuation values varied from −80 to −60 Hounsfield units (HU) in all acquisition phases. Lesion neither dislocate organs nor infiltrate omentum and capsule. Diagnostic refinement has been obtained with contrast-enhanced MRI scan, a 1.5T magnet (Intera 1.5T; Philips Healthcare, Best, The Netherlands) was used to perform MRI examination by a four channels phased array coil. Patient referred to being claustrophobic and in order to reduce the examination time it has been necessary to increase the P-reduction factor (P = 2). The imaging protocol included a *T*_2_ weighted axial (with breath-hold, repetition time [TR] shortest, echo time [TE] 80 ms, slice thickness 7 mm, slice gap 1 mm), *T*_2_ weighted axial spectral presaturation with inversion recovery ( TR shortest, TE 80 ms, slice thickness 7 mm, slice gap 1 mm), *T*_1_ weighted axial (TR shortest, TE 4.6 ms, slice thickness 8 mm, slice gap 1 mm), DUAL (spoiled gradient echo, within and out-phase echoes time; out-of phase: TR shortest, TE 2.3 ms, slice thickness 5 mm, slice gap 1 mm; in-phase: TR shortest, TE 4.6 ms, slice thickness 5 mm, slice gap 1 mm), T2-balance on coronal plane (TR shortest, TE shortest, slice thickness 7 mm, slice gap 1 mm), *T*_1_ high resolution isotropic volume excitation (THRIVE, TR shortest, TE shortest, slice thickness 2 mm at 0^″^ and 25^″^, 80^″^, 150^″^ after administration of contrast medium iv, and a *T*_1_ post Gd (TR shortest, TE 4.6 ms, slice thickness 8 mm). The examination confirmed a well defined mass, with lobulated borders, hyperintense both at *T*_1_ weighted images and *T*_2_ non-fat suppressed images. (Figure 3a, b). DUAL sequences were applied, out-of-phase sequences did not show a signal drop-out (Figure 3c, d), instead *T*_2_ Fat-Sat point out a signal suppression (Figure 3e). THRIVE sequences confirmed an hypointense lesion without significant contrast enhancement (Figure 3f, g). No enlarged lymph nodes has been demonstrated.

## Treatement

Patient has refused to undergo surgery. A core-biopsy has been performed with a 14 G Tru-Cut needle. Histological examination proved that the lesion was a lipoma.

## Discussion

Usually benign tumours of the liver are quite rare, with the exception of cavernous hemangioma which is the most common type of benign lesion that occurs in the liver with an incidence from 0.7% to 7%.^1^ Lipomatous tumours of the liver are very uncommon. Particularly, true lipoma or a mass of predominantly fat tissue, knows as an angiomyolipoma is extremely rare. There are few case series and, generally, this kind of lesion is usually reported as an anthological case in scientific literature more rarely as case series owing to their rarity. An almost complete review of scientific literature shows that cases of true hepatic lipoma are less than 30 cases until 2007, and even if there is an increasing incidence of hepatic benign tumour of the liver, lipoma remains very uncommon.

The first description of primary liver lipoma was only in 1970 when Ramchand et al. described this tumour as incidental diagnosis at autopsy in a 24 years-old male deceased for other causes.^2^

Despite widespread of sensitive imaging studies and increasing report of benign hepatic tumours primary lipoma of the liver is seldom encountered.^3^

Although lipoma can arise from any site, there are only anthological cases dealing with primary lipoma of the liver. Histologically these lesions consist of mature adipose tissue, and have no risk of malignant degeneration.^4^ If small, these kind of tumours are usually asymptomatic and have good prognosis.^5^ Usually the majority of them need no treatment, it is important to discriminate these tumours from malignant ones. The development of this rare benign tumour does not recognize specific risk factors or underlying diseases; however, there is a strong association with fatty involution of the liver parenchyma. In 2012, Martin-Benitez et al. reported a statically significant association between lipomas and non-alcoholic liver steatosis. Hepatic steatosis is generally a diffuse process, but focal distribution of fat, usually periligamentous or periportal, is quite common in the liver and is known as focal fatty changes.^5^ Both pathogenesis of focal fatty changes and primary lipoma of the liver are not clear. Local hypoxia, disturbances in portal flow have been postulated as common cause of fatty changes, but incremented levels of insulin in portal blood seems to be a key factor.^6^ Insulin resistance is responsible for a double alteration in the lipidic metabolism^7^ leading to a greater supply of fatty acids in the liver and restriction within the liver in the form of very low density lipoprotein. A disrupted metabolism could be the underlying cause of both deposit and tumoural entities.^8^ In conclusion, a correlation between liver steatosis, insulin resistance and liver lipoma could be realistic.^9^ Radiologically differential diagnosis could be challenging and atypical features of fatty liver and fat-containing tumours may be a frequent pitfalls of diagnosis. Many diagnostic techniques are impaired by the presence of fat in the liver.^7^ The ultrasound examination of lipomas shows a well-circumscribed uniformly hyperchoic lesion and it could be confused with hemangiomas when small. At CT examination lipomas are homogeneously hypoattenuating masses, with attenuation values raging between −20 to −115 HU, with an increase of density on post-contrast images that does not reach over 30 HU;^5^ when there is a more intense contrast-enhancement it suggests angiomatous components and lesion could be diagnosed as angiomyolipoma. In this case, definitive differential diagnosis is made only on gross specimen, with immune-hystochemical determination of HMB45 (human monoclonal antibody to melanocyte), a surface protein positive in angiomyolipoma and negative in lipoma. MRI scan is the most predictive exam for differential diagnosis. Only lipoma contains fat cells, it should have the same signal intensity as retroperitoneal or subcutaneous fat on all sequences^6^ and provides a signal drop-out on fat-suppressing techniques. Lesions are seen as hyperintense on *T*_1_ weighted images and hypointense on *T*_2_ weighted fat suppressed sequences. Dual echo sequences are pivotal, unlike other imaging modalities, chemical shift MRI scan is a useful technique for confirming the presence of fat in hepatic lipomas. On out-of-phase *T*_1_ weighted gradient echo sequence (GRE), hypointense chemical shift artefacts occur around hepatic lipomas since the signal from fat within a voxel cancels out the signal from water proton in the same voxel. Some MRI scanners have a sequence called “Dixon” which is a *T*_1_ weighted Gradient Recalled Eecho (GRE) or Fast Field Echo (FFE), which allows through a single acquisition to obtain, in the post-processing, a *T*_1_ weighted in-phase, a *T*_1_ weighted out-of-phase, a defined image water (spectral inversion recovery), and a defined image fat (short T1 inversion recovery). Our scanner did not have the use of that sequence.

Other focal liver alterations can show a lipomatous component and must be considered in differential diagnosis (e.g. liver adenoma, hepatocellular carcinoma, angiomyolipoma) but their clinical behaviour is not as indolent as liver lipoma. In particular, liver adenoma is made up by cells filled with glycogen and fat but it has a different behaviour at MR, with hyperintensity In-Phase *T*_1_ weighted GRE sequences and uniform signal loss at Out-Phase *T*_1_ weighted GRE.^6^ Instead, focal nodular hyperplasia appears as a well -circumscribed nodule with intense and uniform enhancement with rapid wash-out associated with a peripheral focal low signal intensity.^6^

## Conclusions

This case report emphasizes the importance of diagnostic imaging, that enables a multiparametric evaluation of several lesions. Diagnosis and characterization of fat in the liver is a diagnostic challenge in radiologist everyday practise. Atypical features of fatty liver and fat containing tumours may be a frequent cause of pitfalls in diagnosis. This case report summarizes the features of the liver lipoma with different imaging techniques.

## Learning points

1. Usually lipoma is a tumour hyperechogenic at ultrasound, but could be also iso-hypoechogenic owing to its heterogeneity, or in a condition of steatosis/fatty liver disease
2. In CT scan, it is hypoattenuating in all acquisition phases, with no increased attenuation during administration of contrast medium. The attenuation values must be less than −20 HU. If the attenuation values increased after infusion of contrast medium you have been considered another kind of lesion similar to lipoma (e.g. angyolipoma and angyomiolipoma).
3. In MRI scan, it appears hyperintense in *T*_1_ and *T*_2_ weighted images, with no drop-out signal in *Out-of-Phase* sequences and hypointense in *T*_2_ fat sat. Usually there is no significant contrast enhancement after administration of contrast medium.

## Consent

An informed consent was obtained from the patient for all the procedures.
